# Is the analysis of territorial compatibility in the vicinity of road hazmat transport routes a necessity for developing countries? A case study of Romania

**DOI:** 10.1016/j.heliyon.2023.e19948

**Published:** 2023-09-09

**Authors:** Andrei Radovici, Horațiu Ștefănie, Iulia Ajtai, Alexandru Mereuță, Camelia Botezan, Alexandru Ozunu, Nicolae Ajtai

**Affiliations:** a “Babeş-Bolyai” University, Faculty of Environmental Science and Engineering, 30 Fantanele Street, 400294 Cluj-Napoca, Romania

**Keywords:** Hazmat, Territorial planning, Developing countries, Road transport, Land use, Risk

## Abstract

The way dangerous goods are transported on roads within the European Union is regulated by international agreements that have been transposed into national legislation. Unlike European policies that focus on preventing major accidents involving dangerous substances in the industry, the component of territorial planning in areas exposed to technological hazards is not given similar attention when it comes to transport routes of dangerous goods. Since both the transport of hazardous materials and the activities of large industrial operators involve the handling of the same types of dangerous substances, they share similar associated hazards. Within this framework, a question arises as to whether decision-makers should accord greater consideration to the land use policies in the areas adjacent to transportation routes. In this study, the case of Romania was analyzed in detail, with the objective to firstly identify some particularities in how the primary road infrastructure is developed in relation to other European countries. Since the territorial compatibility near large industrial operators is established based on specific regulations at the national level, but there is no similar regulation for the transport of dangerous substances, another objective was to adapt and implement a methodology for the analysis of the territorial compatibility in the proximity of the national roads network. The proposed methodology utilizes open-source data and Geographic Information Systems (GIS) for analysis. It also involves the extensive application of existing rules on territorial compatibility for technological risks, specifically in the context of hazmat transport. The results of this study indicate that the primary road transport network in Romania has different characteristics compared to that of other countries, which determines a higher level of exposure to the hazards specific to the road transport of hazardous materials. Additionally, from an analysis of gasoline and diesel transport on national roads and the implementation of a territorial compatibility matrix, we observed instances of territorial incompatibility in the current state, particularly in areas close to the road, especially for scenarios with a relatively high accident frequency. Thus, the way future large road infrastructure development projects address risks related to dangerous goods transportation and the implementation of environmentally conscious land management strategies can contribute to society's sustainable development.

## 1 Introduction

Global trends in the quantity of hazardous materials (hereafter under the name of hazmats) transported by road vary, but there has generally been an increase in such transport in recent times [[1], [2], [3], [4]]. This is particularly true for developing countries, where the demand for hazmat is growing at a faster rate than in developed countries [5]. It was observed that in the most developed countries, there is a trend towards a modal shift [6], where hazmats are transported by rail or water instead of road. This can be driven by a number of factors, including environmental concerns, cost savings, and the capacity of existing transport infrastructure. According to the United Nations Development Programme, Romania is ranked as a developed country (53rd globally) based on the 2021 Human Development Index [7]. However, Eurostat data shows that the road infrastructure is still in development-among the lowest density of highways in EU [8].

According to the World Health Organization, the transport of hazmats by road has been steadily increasing worldwide, with an average annual growth rate of 2.8% between 2010 and 2017 [9]. This growth has been driven primarily by the demand for petrochemicals used in various industries, including energy, chemicals, and plastics [10]. In addition, many hazmats are required for essential industries, for example healthcare, and the pandemic has increased the demand for specific hazmats, such as medical gases [11].

The scientific literature clearly specifies that hazmats can pose a significant risk to public safety in the event of an accident or spill [[12], [13], [14], [15], [16], [17]]. Besides public safety, hazmats can also pose a risk to the environment [18]. Assessing the risks associated with the transportation of hazmats facilitates the identification of high-risk areas and the implementation of measures aimed at minimizing risks, consequently enhancing public safety. Moreover, measures can be put in place to minimize the environmental impact of such incidents.

In earlier research focused on the examination of the hazmat transportation network at either a local or regional level within Romania, the authors determined that many transport routes pass through densely populated areas, including villages, towns, and even cities [19]. This fact should have significant implications in terms of territorial planning, both for the existing infrastructure elements and especially for the future ones. This consideration also led to the research hypothesis for this study, namely that the current state of the road infrastructure in Romania (characterized by low density of highways and bypasses) generates a high level of exposure to the hazards related to the transport of hazmats at national level, and is not specific only to a local or regional level.

As hazardous material transportation occurs in populated areas, it significantly raises risks for people and the environment due to increased exposure and higher potential dangers [20]. In this context, it's important to conduct an analysis from which it can be seen whether the results of the studies are relevant only for the geographical area analyzed, or if they are valid at national level. Furthermore, the analysis results can be used in developing land use policies near road transport routes of hazmats. These policies can be crucial for public safety, environmental protection, and risk management overall.

Land use policies can be used to ensure that residential, commercial, or other sensitive land use types are not located too close to road transport routes of hazmats. For example, policies should prohibit or restrict the construction of schools, hospitals, or residential buildings within a certain distance of the transport routes. This can help to reduce the potential for harm to people in the event of an accident or spill. Land use policies can also be used to facilitate risk management efforts by ensuring that emergency responders have easy and safe access to hazmat transport routes [21]. Especially in Romania, policies should also require the construction of highways in order to take over most of the hazmat traffic that currently takes place on national roads.

Currently, there are no legislative provisions in Romania aimed at territorial compatibility in the vicinity of hazmat transport routes. In more developed countries, like the United States, the Department of Transportation (DOT) regulates the transportation of hazmats through its Pipeline and Hazardous Materials Safety Administration (PHMSA) [22]. PHMSA has established regulations that require local governments to develop and enforce land use plans for areas around hazmat transportation routes, known as “Transportation Safety Zones” or “Hazardous Materials Route National Standard” depending on the mode of transportation. In Canada, the Transportation of Dangerous Goods Act and Regulations, administered by Transport Canada, sets out requirements for the transport of hazmats, including the establishment of safety zones around transportation routes [23]. In the United Kingdom, the Control of Major Accident Hazards Regulations (COMAH) [24] requires companies that handle hazmats to assess and manage the risks of major accidents and to ensure that land use around transportation routes is compatible with the risks associated with hazardous materials. Germany is another example of a country that regulated the handling of hazmats through its Federal Immission Control Act, including establishing safety zones around transportation routes and the requirement for emergency response plans [25]. In a trans frontier cooperation project between Italy and Switzerland, focused on “Monitoring the transport of dangerous goods as a means of protecting the territory,” an information system was developed and implemented. The main objectives of this system were to monitor, collect, and analyze data to effectively quantify and manage the risks linked to transporting hazardous materials by road [26,27]. The platform publicly provided data concerning hazmat transports to interested parties, enabling them to analyze, process, and share the information [26]. The system's applications have proven beneficial for land and transport planning processes, including implementing restrictions on hazmat transport vehicle transit and facilitating routing and re-routing actions [26]. However, it is unfortunate that such a highly valuable approach cannot be implemented in Romania within a publicly available system. This limitation arises from the fact that data related to hazmat transports are classified as confidential and are exclusively accessible to environmental protection agencies and inspectorates for emergency situations.

Scientific literature highlights multiple approaches to assess the issues related to the transportation of hazmats, but in general, there are two main perspectives focused on route planning [[28], [29], [30]] and historical accident data mining [2,[31], [32], [33]]. As Ma et al. stated, the process and method of risk assessment in these types of studies are not always in focus [34]. Many of the models used in these studies depend on the probability of an accident and the size of the consequences [35]. In a comprehensive overview of relevant literature, Mohri et al. conclude that mainly the population included in the radius or bandwidth of the affected area around the transportation routes is used for estimating the consequences, but the impact on other exposed elements should also be considered [36].

A study carried out in the Netherlands, generated a hypothesis similar to this study, starting from the fact that rail transport of hazmats in the Netherlands is organized in a manner similar to the road network in Romania (right through city centers)- fact that creates a strong link between rail transport, urban planning, and the development near railways [37]. One of the study's main conclusions was that although the policymakers responsible for transportation considered improving railway safety, systematizing a consequence-based approach for land use planning will largely contribute to risk reduction [37].

Taking into consideration the international research panorama described earlier, the innovative contribution of this research lies in tailoring an approach to the unique context of developing countries and providing valuable recommendations for improving hazmat transportation practices in these regions. This study aims to demonstrate how open data sources can be effectively utilized to increase the possibility of conducting similar studies in other developing regions by promoting transparency and replicability. Also, by aligning the proposed methodology with existing legislated provisions and regulations relevant to hazmat transportation, the authorities may be more receptive in adopting the proposed policy changes, while increasing the likelihood of successful policy implementation and long-term impact. This paper introduces another novel aspect by conducting a comparative analysis of Romania's hazmat transportation practices, focusing on the current state of the infrastructure, with those of other developing countries or even developed nations. This comparative approach aims to emphasize potential gaps and identify areas for improvement in hazmat transport management and safety measures.

In summary, assessing the risks related to the transport of hazmats is crucial for public safety, environmental protection, compliance, and risk management purposes. It is essential that companies and regulatory agencies work together to ensure that appropriate risk assessments are conducted and that measures are put in place to minimize the risks associated with hazmat transportation. In this context, the main objectives of this study are twofold. Firstly, we aim to develop a methodology for conducting a comparative analysis of road transport network characteristics between Romania and other European countries. Secondly, we seek to create a comprehensive methodology in order to analyze the territorial compatibility in the proximity of the primary road transport network using GIS tools and leveraging free and open-source data. The ultimate goal is to gain valuable insights into the level of exposure to hazards related to hazardous materials (hazmat) transport in Romania. By applying GIS tools and utilizing freely available data, we aim to contribute to a better understanding of potential hazards associated with hazmat road transport in the country.

The following sections of the article were structured ensuring clear division of their respective contents. Section 2 focuses solely on presenting the methodology used in the study, and it is divided into two parts. The initial section outlines the steps to compare land use around major road transportation infrastructure. This analysis is meant to provide insights into how Romania's primary road network development compares to other countries. The second part describes the steps in establishing territorial compatibility for hazmat transport routes, covering topics such as determining impact zones, functional areas, and the application of the compatibility matrix. Section 3 is dedicated to the results and discussion, following the same two-part structure as Section 2. It showcases the outcomes of the applied methodology, offers a comparison with similar issues discussed in the literature, and includes an analysis of the study's limitations and strengths. Finally, in Section 4, the article concludes by presenting the key findings and implications drawn from the research.

## 2 Materials and methods

### 2.1 Comparative analysis regarding the land use in the vicinity of the primary road transport infrastructure

A comparative analysis regarding the road infrastructure in several EU states was carried out to understand better how the primary road transport network in Romania was developed compared with other states. As a result, specific characteristics of the Romanian transport network have been identified. The first stage of this analysis was to overlap the layer containing the primary road network information (Open Street Map) [38] with a layer containing information on land use at the national level, using GIS analysis software. The main advantages of this database are unrestricted access, the amount of available data, and the fact that it is constantly updated. Data regarding the land use type in Romania was extracted from the Corine Land Cover database [39], a database available for all member states of the European Union [40]. By overlapping the two layers, a statistical ratio of the land use types near the national roads in Romania was developed. The same analysis was undertaken for other UE states in the next stage. The states that were included in the comparative analysis were selected considering the availability of similar data and the developing level of the states. This last criterion aimed to include in the analysis the states with a similar level of road infrastructure development as the one in Romania, as well as some with a more developed infrastructure.

### 2.2 Establishing the territorial compatibility for hazmat transport routes

The approach to determining territorial compatibility for hazardous materials transportation relies on the regulations stipulated by Romanian legislation in the Order no. 3710/1212/99/2017- regarding the approval of the methodology for establishing adequate distances from potential sources of risk within the sites that fall within the provisions of Law no. 59/2016 on the control of major accident hazards involving dangerous substances in land use planning and urban planning activities [41]. This law aims to establish adequate distances from the so-called Seveso sites, in case of major accidents in which hazardous substances are involved, both for the construction of new sites and for changes made to existing sites. Furthermore, the law also covers the new constructions near the previously mentioned sites.

As the dangers linked with hazmat transportation resemble those originating from industrial operations, the criteria for defining and visually depicting impact zones along transportation routes have been adjusted and utilized. Hence, the current approach aims to pinpoint the main national routes for road-based hazardous material transportation, along with the substances commonly carried. As in the case of Order 3710/2017, the determination and representation of the impact zones are done in accordance with the potential effects of an accident on the population. In this context, the method used in this study for the territorial compatibility analysis is based on consequences. This approach has its advantages and limitations, which are being treated exhaustively by Török et al. [42], and substantially differs from risk-based methods [[43], [44], [45]].

Thus, using this methodology, three impact zones were identified according to the threshold values for specific effects, as follows.

• Zone I - High lethality area;
• Zone II – The beginning of the lethality area;
• Zone III – Irreversible effects area.

The current methodology for analysis proposes the exclusion of regions that exclusively result in reversible injuries, primarily due to the relatively lower frequency of hazardous substance transportation accidents in comparison to Seveso sites. Because the accident frequency value in each scenario dictates the suitable type of functional zones, selecting the reversible effects zone would have led to compatibility in all situations.The impact zone delineation and the modeling of the effects produced by an accident is done based on the threshold values presented in Table 1. The effects were modeled considering the worst credible scenarios for each type of transport.

**Table 1:** Threshold values for specific effects on the population (adapted after OM 3710/2017).

Hazard Type	High lethality area	The beginning of lethality area	Irreversible effects area
Toxic dispersion	LC 50	AEGL 3	AEGL 2
Fire	12.5 kW/m^2^	7 kW/m^2^	5 kW/m^2^
Explosion	30 mbar	14 mbar	7 mbar

*LC 50- Lethal concentration of a substance in the air required to kill 50% of the people exposed.

AEGL 3- Life-threatening health effects or death.

AEGL 2- Irreversible or other serious, long-lasting adverse health effects or an impaired ability to escape.

The CORINE Land Cover database was used to delineate the functional areas near the road transport routes of dangerous substances. In order to determine territorial compatibility, it was essential to allocate different degrees of vulnerability to land use classes or types and categorize them into distinct functional zones. The vulnerability of the exposed territorial elements is expressed qualitatively, and it's taking into account the safety standards for population, infrastructure, and environment. Depending on the vulnerability degree, the territorial elements must fall into the following functional areas:

**Zone A** - slightly vulnerable territorial elements in the event of a transport accident involving hazmats. This category includes those land use types characterized by the lack of built anthropic elements or by biotic or abiotic components that would not be significantly affected by the effects of an accident.

**Zone B** - territorial elements with a medium vulnerability in the event of a transport accident involving hazmats. This category includes those land use types characterized by a limited presence of anthropogenic elements or, where they exist, the effects of an accident would produce material losses in particular. The effects of an accident can affect the biotic and abiotic components of the ecosystem.

**Zone C** - territorial elements with a high vulnerability in the event of a transport accident involving hazmats. This category includes those land use types characterized by a significant density of anthropogenic elements (residential areas, playgrounds, green spaces, transport infrastructure, etc.) and population. The effects of an accident would produce both material and human losses as well as the disruption of social activities.

**Zone D** - territorial elements with a very high vulnerability in the event of a transport accident involving hazmats. This category includes those land use types characterized by a very high density of anthropogenic elements (dense residential areas with a high height regime, areas where critical infrastructures relevant at the national level are present, constructions with a particular regime, etc.), and population. The effects of an accident would produce significant material and human losses as well as disruption of social activities and exposed ecosystems.

To assign values for accident frequencies, the specific literature was consulted. The accident frequency values resulted from the analysis of historical accidents produced in the United States of America for 5 decades were used [46]. Based on this historical analysis, it was established that the highest value of the accident rate characteristic for the transports of flammable gases is 1.12*10^−6^ acc./km/year.

Finally, in order to determine the territorial compatibility the following steps are necessary: i) the overlapping of the impact areas in the vicinity of the transport routes of hazmats with the territorial elements registered in the Corine database; ii) the classification, considering the level of vulnerability, in one of the four functional areas previously described. This stage is followed by applying a compatibility matrix (Table 2) specific to the accident frequency for the given scenario.

**Table 2:** The matrix used for establishing territorial compatibility for hazmat transport routes.

Frequency (accidents/route/year)	Impact areas		
	Zone I – High lethality	Zone II - The beginning of lethality	Zone III - Irreversible effects
10^−3^ -10^−4^	A	A	A
10^−4^ -10^−5^	A	A	AB
10^−5^ -10^−6^	A	AB	ABC
<10^−6^	AB	ABC	ABCD

To implement the territorial compatibility matrix, a sequence of operations for generating, analyzing, and editing the land use data was required, with the purpose of data integration into the GIS software. Therefore, several steps were followed in the preparation of the data.

The first step is to model the effects and the consequences of the accidents in which hazmats are released. The *Effects* modeling software (v10), developed by the Netherlands Organization for Applied Scientific Research (TNO) company, was used for the modeling. In order to obtain values of the characteristic distances for each of the three thresholds provided in the methodology, a set of input data are needed: data regarding the properties of the hazmats, the means of transport, the accident scenario, and the weather conditions.

**Fig. 1:**
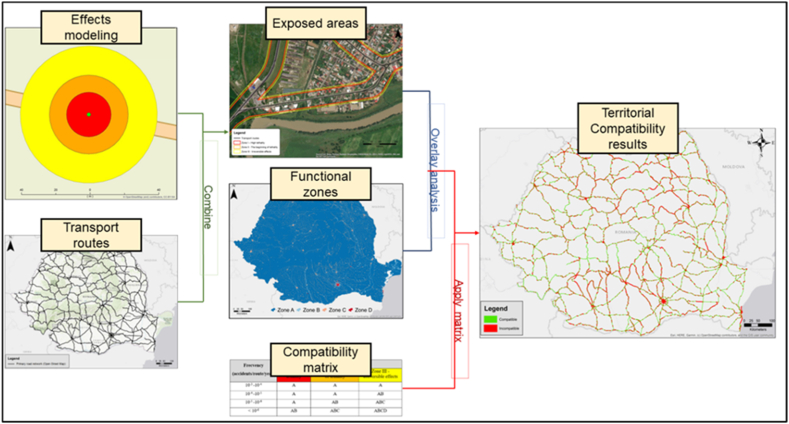
The methodological framework for establishing territorial compatibility.

Moreover, since the representation of the impact areas is done along the transport routes, it is necessary to use a vector file containing the transport routes or the road transport network. These files were extracted from the Open Street Map database [38]. Finally, the information regarding the land use types in the impact areas is extracted using GIS techniques. Once new files (containing the land use data for each impact area) are generated, the territorial compatibility can be determined based on the matrix described in this methodology. An outline of the methodological framework is presented in Fig. 1.

## 3 Results and discussion

### 3.1 Comparative analysis regarding the land use in the vicinity of the primary road transport infrastructure

**Fig. 2:**
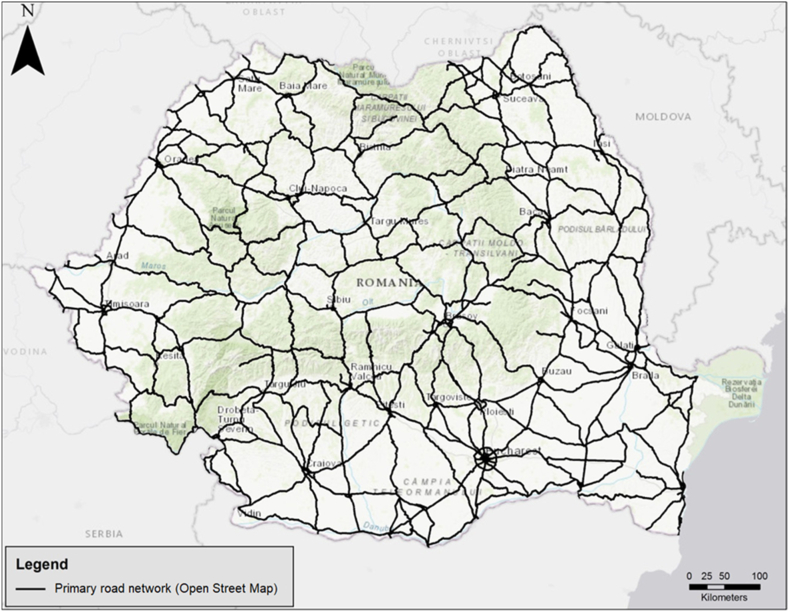
Romanian primary road network included in the analysis.

As stated in the methodology section, the data were retrieved through the Geofabrik.de portal, which allows access to data from the OpenStreetMap project updated daily. Only the fields with the representative codes for the national roads and their access roads were extracted from the database. The files for highways were not included in the analysis, as they are poorly represented, and they do not incorporate a significant part of the hazmat traffic in Romania. In order for the results of the analysis to be comparable, the method of data extraction is similar for the other countries included in the analysis. By querying and extracting the data from the previously mentioned sources, it was possible to create a file containing information about the Romanian primary road network (National Roads), as seen in Fig. 2.

Further on, the process involved superimposing the national road network from the OpenStreetMap inventory onto a dataset with land use details (CORINE inventory). This allowed for the retrieval of information regarding the primary land use categories intersected by Romania's National Roads Network. (Table 3).

**Table 3:** The main land use classes crossed by the national roads in Romania.

CORINE code	Nomenclature	Percentage of the total length of the roads (%)
112	Discontinuous urban fabric	34.5
211	Non-irrigated arable land	30.6
311	Broad-leaved forest	5.7
242	Complex cultivation patterns	5.6
231	Pastures	5.5
121	Industrial or commercial units	4.6

The data provided in Table 3 support the idea that a significant portion of the primary road transportation routes in Romania either pass through towns or are situated very close to them as the discontinuous urban fabric and the industrial or commercial units are found among the most prevalent classes. The fact that the second most common class is represented by agricultural land may also be of interest since, according to the Corine and Urban Atlas inventory, in the 2012–2018 interval, agricultural land was mainly converted into residential areas, especially on the outskirts of cities [47,48]. If the current situation of the road infrastructure will not improve and this changing trend in land use will continue, we will observe in the future even more vulnerable areas that will be exposed to the hazards associated with hazmat transport.

To determine if the considerable proportion of primary road network traversing through towns is unique in Romania or is a common occurrence in other states, a range of countries were chosen for comparison: Bulgaria, Austria, Croatia, Spain, the Netherlands, Hungary, and Slovakia. Although the road infrastructure (highways) in all the countries included in the analysis is much more developed than in Romania, two large categories can be distinguished: countries with a well-developed highway network (Netherlands, Spain, Croatia, Austria) and countries with a highway network in development (Hungary, Slovakia, Bulgaria).

Results regarding the main categories of land use crossed by the primary road network show a significant discrepancy in how the road network in Romania is developed compared to the other cases: most of the infrastructure is developed in built-up areas with little or no alternatives for routing the transport on highways (Table 3). On the other side of the spectrum is the case of Spain, where only 8.5% of the primary road network is flanked by lands included in class 112- „Discontinuous urban fabric.” Values similar to Romania were obtained in Austria, where almost 31% of the national roads are disposed near localities. This fact can be attributed to the limitations imposed by the topography. However, it should be mentioned that in the case of Austria, the highway network is very well developed, and most transports take place far from inhabited communities and/or densely populated areas. For the other countries, the values were between 14% in Bulgaria and 21% in Croatia for the previously mentioned land use class.

### 3.2 Establishing the territorial compatibility for hazmat transport routes

Taking into account the fact that the road transport of flammable liquids represents over 50% of the entire transport of hazmats in Europe [49,50], we conducted the territorial compatibility analysis for these scenarios.

**Fig. 3:**
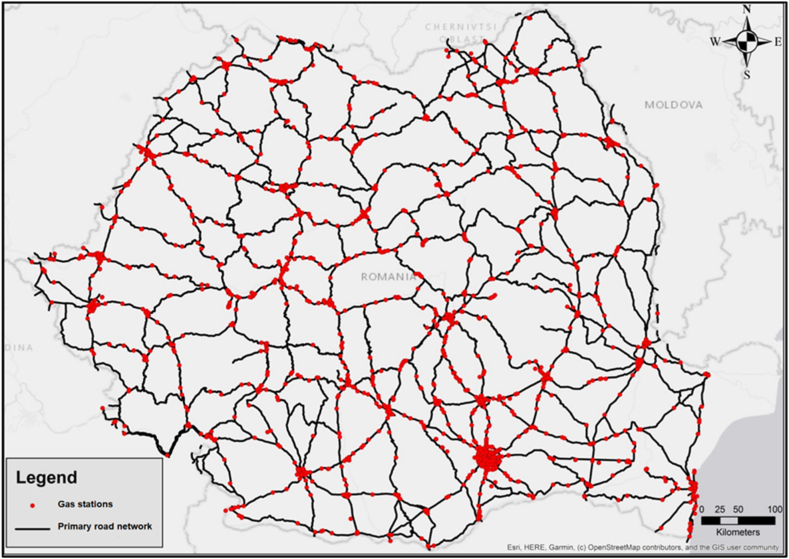
Distribution of gas stations in the vicinity of Romania's national roads.

The OpenStreetMap provides information concerning the whereabouts of different locations, like gas stations. To assess the viability of the entire national road network for gasoline and diesel transportation, we identified nearby gas stations within a maximum range of 5 km. This value was chosen based on the fact that all gas stations located at a smaller distance will predominantly use the primary road transport network for supply. Drawing insights from the examination of the spreading of gas stations (Fig. 3) concerning Romania's national roads, it can be affirmed that the distribution holds a consistent pattern, with nearly all key road infrastructure being utilized for fuel transportation. Starting from these findings, it was decided that the territorial compatibility analysis would be implemented at national level for the entire primary road network. To approach other hazmats in this type of analysis might be difficult because the data related to the routes and the transported quantities are confidential and not disclosed.

After conducting simulations of the potential physical repercussions on the population (utilizing worst-case accident scenarios involving diesel and gasoline transport tanks), the results are outlined in Table 4. The analyzed scenarios assume the transport of 22 tons of fuel as it is the maximum quantity allowed in our country. For the transport of gasoline, the thermal radiation from fire and the overpressure caused by the explosion were modeled. For diesel, the effects of heat as a result of a failure in the tank and the ignition of the entire quantity of cargo were modeled.

**Table 4:** Threshold distances of the physical effects on population.

Transport type	Hazard	High lethality threshold (m)	Lethality threshold (m)	Irreversible effects threshold (m)
Gasoline	Fire	14.5	25	52
	Explosion	45.2	48.2	56.3
Diesel	Fire	13	23	40

Once the specific distances for each area were calculated, perimeters were delimited along the national roads to extract the data related to land use. As described in the methodology section, a distinct score corresponding to the level of vulnerability was attributed to each land use class. This categorization determined its placement within one of the four types of functional zones (Table 5). This type of reclassification was carried out in other similar studies [17,42], and can be used for different land use inventories with a similar structure.

**Table 5:** Land use classes grouping into categories of functional areas based on the degree of vulnerability.

Type of functional area	Corine land use classes	
A (Low vulnerability)	Mineral extraction sites	Dump sites
	Non-irrigated arable land	Permanently irrigated land
	Rice fields	Vineyards
	Fruit trees and berry plantations	Pastures
	Annual crops associated with permanent crops	Complex cultivation patterns
	Land principally occupied by agriculture, with significant areas of natural vegetation	Broad-leaved forest
	Coniferous forest	Mixed forest
	Shrub and/or herbaceous vegetation associations	Transitional woodland/shrub
	Beaches, dunes, sands	Bare rock
	Sparsely vegetated areas	Inland wetlands
B (Medium vulnerability)	Industrial or commercial units	Construction sites
	Water courses	Water bodies
	Sea and ocean	
C (High vulnerability)	Discontinuous urban fabric	Road and rail networks and associated land
	Green urban areas	Sport and leisure facilities
D (Very high vulnerability)	Continuous urban fabric	Port areas
	Airports	

**Fig. 4:**
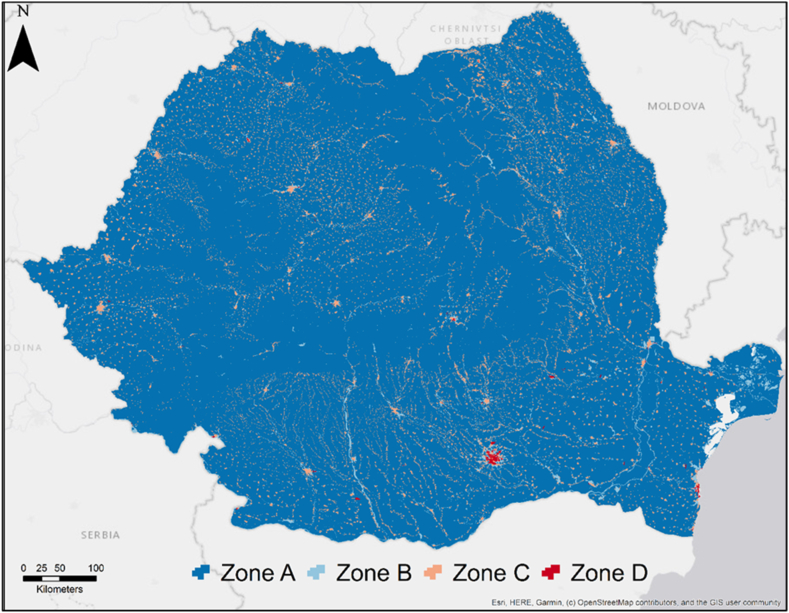
Functional areas in Romania (Corine land cover reclassified).

The high degree of population density and anthropic elements coverage (residential areas, infrastructure etc.) determined the inclusion of the continuous urban area in the class characterized by the highest vulnerability (Zone D), according to the functional areas described in the methodology. Port areas and airports were also included in the same class, as they represent critical infrastructures at national level, and any disruption of their activity has negative consequences in different fields of activity. The classification of the Romanian territory into functional zones can be observed in Fig. 4.

By overlying the impact zones near the hazmat routes (Table 4), resulted from the modelling, with the elements present in the Corine inventory (Fig. 4) and applying the compatibility matrix (Table 2), it was possible to develop the territorial compatibility analysis for national road network in Romania used in the transport of gasoline and diesel.

The compatibility analysis reveal concerns about territorial alignment between Romania's primary road network and neighboring regions regarding the relation to hazmat transportation - specifically, gasoline and diesel in this instance. Furthermore, when analyzing the specific accident frequency class for most of the fuel transports routes (between 10^−5^ -10^−6^ events/route/year), the data presented in Table 6 was obtained. This frequency range was obtained by multiplying the basic frequency specific to each hazmat class with the potential length of the transport route. Consequently, in order to fall within this range, the routes must have a length between 25.7 and 257 km for fire scenarios and 51–510 km for explosion scenarios. From the public data regarding the distribution infrastructure of a Romanian fuel producer [51], it appears that the primary transport of fuels from the refinery to the regional warehouses takes place by rail, only the final distribution to the gas stations is done by road. This aspect determines shorter transport routes (generally shorter than 250 km), whose frequency falls within the previously mentioned interval.

**Table 6:** Synthesis of territorial incompatibility resulted for the transports with an accident frequency of between 10^−5^ -10^−6^ events/year.

Impact areas	Substance	Associated hazard	Incompatible areas percentage
Zone I – High lethality	Gasoline	Fire	41.6%
		Explosion	41.7%
	Diesel	Fire	43%
Zone II - The beginning of lethality	Gasoline	Fire	36%
		Explosion	34.1%
	Diesel	Fire	36.2%
Zone III - Irreversible effects	Gasoline	Fire	1%
		Explosion	0.9%
	Diesel	Fire	0.9%

**Fig. 5:**
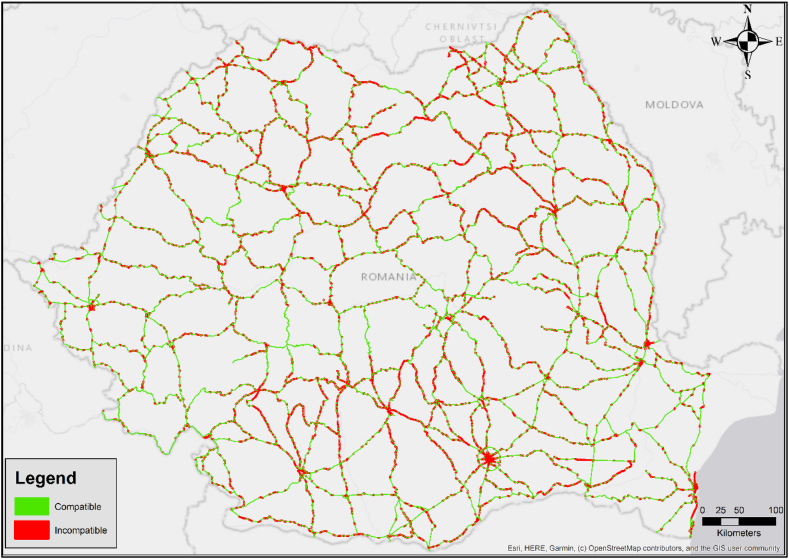
Graphical representation of the results of territorial compatibility analysis for gasoline transport on Romanian national roads (Zone II: accident frequencies between 10^−5^ -10^−6^ events/year).

Data in Table 6 show that over 40% from the areas included in the high lethality zone presents territorial incompatibilities with hazmat transport activity, and more than one third of the areas in the beginning of lethality zone are in the same situation. The situation changes significantly for the areas further away from the transport routes (those corresponding to areas with irreversible effects), under one percent of these areas being in territorial incompatibility. An example of a graphical representation of one case is presented in Fig. 5. However, this figure is only illustrative of the situation at national level; the contours of the areas in territorial compatibility or incompatibility are not being generated to scale (the size of the contours is greatly exaggerated).

Therefore, the results regarding the spatial distribution of the incompatible areas within the three impact areas considered in this study emphasize the necessity of methods and tools that will reduce the impact of hazmat accidents and will ensure safe transportation.

Considering the increasing risk of hazmat accidents during transportation in the past years [4] and the results of this study regarding the territorial incompatibility in Romania, the improvement of territorial planning and the legislative regulation of hazmat transportation should be a priority for the interested parties. This is a pressing issue in Romania due to the fact that most transportation roads cross inhabited areas (as the results of this study showed), the hazmat accidents having the potential to generate great consequences. Moreover, the rapid urbanization may increase the exposure of certain communities to this hazard, in areas where the territorial planning is not well adapted and does not take into account the potential risks related to hazmat transportation. In this context, properly designed emergency management plans and procedures can increase the resilience of the exposed elements. This was also emphasized in a study focusing on the resilience of road network infrastructure as critical infrastructure, where Borghetti et al. assessed in detail the capacity of operators and first responders to intervene in case of an event with possible disruptive consequences [52]. The fact that, in Romania, the Inspectorate for Emergency Situations is the institution responsible for issuing authorizations for hazmat transports on approved routes and also for intervening in the event of an accident, is beneficial because through a comprehensive evaluation of its response capabilities in various areas, the authority can decide to select a route with a higher exposure level, considering the presence of a stronger response capacity. On the other hand, the authority might consider a route unsuitable for hazmat transportation if it lacks sufficient response capabilities as any incident on that route could lead to severe consequences without proper and timely intervention. Even this aspect of organizing the response capabilities in order to provide timely intervention is influenced by the way the road network is developed. Generally, the existence of a well-developed road infrastructure facilitates the access of intervention teams to the place where the accident occurred.

Another issue discussed in the literature [28] is the one related to the equitable risk distribution among the population, which calls for an optimization of the transport process. However, the routes choice may prove a difficult task that must take into account the exposed communities and the potential consequences in case of accidents (regarding the population as well as the environment). In other words, the practitioners must consider the local context and therefore, the procedures must be adapted to specific situations. This fact is particularly relevant in countries with a developing infrastructure, such as Romania, where the route choices are limited because the highway network is poorly developed. The development of road infrastructure in Romania, particularly the highway network, is significantly and positively influenced by its membership status in the European Union. The majority of post-accession road infrastructure investments have been facilitated through European funds. Looking ahead, the National Recovery and Resilience Plan [53], also funded by the EU, outlines ambitious goals, including extending the highway network by over 400 km by 2026 [54]. Moreover, the plan emphasizes the modernization of other transport sectors, including the railway system. These factors present a unique opportunity for Romania, as a country with developing infrastructure, to enhance overall resilience and safety in hazmat transport.

Regarding the strengths of this study, one notable aspect lies in the utilization of open-source data for analyzing two major components: firstly, the extraction of geospatial data concerning the transport infrastructure, and secondly, the establishment of functional areas based on a Corine inventory accessible in 39 countries. This approach establishes a groundwork for potential development of similar studies in various geographical regions and with varying spatial extents. Furthermore, it is worth noting that both modeling physical effects and conducting analyses through GIS techniques can be accomplished using freely available software packages like Areal Locations of Hazardous Atmospheres (ALOHA) for hazard modeling and QGIS for the analys of geospatial data. An additional advantageous aspect of the proposed methodology pertains to its final products, specifically the territorial compatibility maps. These maps are readily comprehensible even to non-specialists, including NGO representatives, interested parties, economic operators, and others. Moreover, they can be effortlessly integrated into development plans and policies, facilitating their practical application. The integration of this approach, particularly in the context of Romania, is facilitated by its reliance on established criteria, thresholds, and methods that are already legislated for a similar sector with regards to associated hazards. On the other hand, there are also very important limitations related to the application of the proposed methodology. These limitations manifest in various directions. Primarily, the analysis method is consequence-based (or deterministic), and as such, it does not directly quantify the likelihood of selected accident scenarios. Instead, it relies on expert judgment, historical data, and hazard analysis for scenario selection. While risk-based (or probabilistic) methods are more complex and require a larger input data set, they yield more realistic results concerning potentially affected areas [42]. provides a comprehensive analysis of the disparities between these two approaches in analyzing territorial compatibility for SEVESO sites. Secondly, it is worth noting that this method of establishing territorial compatibility is more suitable for scenarios resulting from fires or explosions and less suitable for scenarios resulting from toxic dispersions. This is because the modeling of toxic dispersions for different substances does not account for topography, leading to smaller distances but higher concentrations in valley areas. An essential source of uncertainties in applying the methodology to an extended area lies in the weather-related data used in the calculation of physical effects. In reality, average weather conditions vary significantly in different regions, particularly influencing the analysis of transports involving substances that can generate toxic dispersions in the atmosphere. In contrast, the modeling of fires and explosions is less dependent on weather conditions. One final limitation of this methodology lies in its dependence on the resolution of the database used for determining functional areas, as the accuracy of establishing territorial compatibility is directly proportional to this resolution. While the present approach utilizing the Corine inventory is advantageous for large-scale analyses at the national or regional level, its low resolution hampers detailed assessments at the local level, particularly in heterogeneous areas. As an alternative, to determine the functional areas at the local level based on the criteria outlined in the methodology, the Urban Atlas inventory (where available) or urban plans developed by local authorities may be utilized.

## 4 Conclusions

At the international science community level and within the developed state governments, there is an active attitude concerning land use planning, considering hazmat transport. This concern is transposed in the identification of alternative routes and choosing the safest ones for the transport of hazmats based on population density, road category, the type of the transported hazmat and the quantity, response capacities, routes continuity, economic impacts, weather conditions, and accidents history. However, due to Romania possessing a road infrastructure that inadequately accommodates the present demands of both passenger and freight transportation, territorial planning policies cannot solely focus on selecting the most optimal routes. In numerous instances, alternatives are simply unavailable.

Considering that all the primary roads are used for hazmats transport, which is backed up by the analysis made for the fuel transport, the territorial planning must focus on two main directions: development of a highway network and the restriction of land use changes (in the hazard occurrence areas) near the primary roads.

The outcomes of the initial phase in the analysis reveal that Romania's national road network predominantly passes through urbanized regions, exhibiting the greatest percentage among all the countries under examination. This aspect leads to an increased vulnerability of susceptible elements to the risks linked with hazmat transportation.

The analysis of territorial compatibility near road hazmat transport routes is a necessity in developing countries, where the land-use planning is still not adapted to the rapid grow of population and urbanization. The proposed work methodology is flexible enough to allow its application in other cases as well.

The results obtained indicate that policymakers need to enforce sustainable development strategies that restrain the vulnerability of exposed components in close proximity to routes used for transporting hazardous substances. A better approach to minimize exposure involves establishing alternate routes. While the frequency and impacts of accidents involving hazmat-carrying vehicles cannot solely substantiate the need for significant infrastructure projects, they could play a crucial role in the overall cost-benefit assessment. These considerations, alongside economic viability, could encompass factors like pollution reduction in cities and accident reduction. The territorial planning process near the transport routes of hazmats should be managed by a committee, including the authorities responsible for territorial development. Furthermore, in case of incompatibilities for existing or future routes and vicinities, the responsible authorities must require the implementation of necessary risk reduction measures like speed limitations, improve response capacities, warning communication and dissemination systems to reach the entire exposed population. The municipalities on whose administrative territories are located in impact areas may use the compatibility plans when issuing urban certificates and building permits.

The final conclusion that can be drawn from this study is that despite its deterministic approach, wherein a series of methodological steps were adapted, it effectively demonstrates the functionality of applying a simple methodology while utilizing widely available data across an extended area in order to highlight the existing problems in the territorial planning policies implemented so far in a country experiencing ongoing infrastructure development. However, future research must focus on developing a more optimized probabilistic analysis method with criterions based on individual risk contours and social risk curves. This method should be applied to smaller areas or better-documented transport routes. Additionally, it should take into account a series of crucial elements in establishing territorial compatibility, such as minimum cost and minimum risk paths, accident frequencies, response capabilities, etc.

## Funding

This work was supported by 10.13039/501100006347Babeș-Bolyai University of Cluj-Napoca, grant no AGC36539/November 25, 2022 and grant no AGC36440/November 21, 2022.

## Author contribution statement

Andrei Radovici: Conceived and designed the experiments; Performed the experiments; Analyzed and interpreted the data; Wrote the paper.

Horatiu Stefanie: Conceived and designed the experiments; Analyzed and interpreted the data; Wrote the paper.

Iulia Ajtai: Performed the experiments; Analyzed and interpreted the data.

Alexandru Mereuta: Performed the experiments; Contributed reagents, materials, analysis tools or data.

Camelia Botezan: Performed the experiments; Wrote the paper.

Alexandru Ozunu: Nicolae Ajtai: Conceived and designed the experiments; Contributed reagents, materials, analysis tools or data.

## Data availability statement

Data will be made available on request.

## Declaration of competing interest

The authors declare that they have no known competing financial interests or personal relationships that could have appeared to influence the work reported in this paper.
